# Quantitative Analysis of Tau-Microtubule Interaction Using FRET

**DOI:** 10.3390/ijms150814697

**Published:** 2014-08-21

**Authors:** Isabelle L. Di Maïo, Pascale Barbier, Diane Allegro, Cédric Brault, Vincent Peyrot

**Affiliations:** Aix-Marseille Université, Inserm, CRO2 UMR_S 911, Faculté de Pharmacie, 27 Bd Jean Moulin, 13385 Marseille, France; E-Mails: pascale.barbier@univ-amu.fr (P.B.); diane.allegro@univ-amu.fr (D.A.); cedric.brault@free.fr (C.B.)

**Keywords:** tau, microtubules, FRET, energy transfer, interaction

## Abstract

The interaction between the microtubule associated protein, tau and the microtubules is investigated. A fluorescence resonance energy transfer (FRET) assay was used to determine the distance separating tau to the microtubule wall, as well as the binding parameters of the interaction. By using microtubules stabilized with Flutax-2 as donor and tau labeled with rhodamine as acceptor, a donor-to-acceptor distance of 54 ± 1 Å was found. A molecular model is proposed in which Flutax-2 is directly accessible to tau-rhodamine molecules for energy transfer. By titration, we calculated the stoichiometric dissociation constant to be equal to 1.0 ± 0.5 µM. The influence of the *C*-terminal tails of αβ-tubulin on the tau-microtubule interaction is presented once a procedure to form homogeneous solution of cleaved tubulin has been determined. The results indicate that the *C*-terminal tails of α- and β-tubulin by electrostatic effects and of recruitment seem to be involved in the binding mechanism of tau.

## 1. Introduction

Tau is the major microtubule associated protein (MAP) in neuronal axons [1] that stabilizes microtubules and maintains neuronal process [2,3]. Microtubules, generated by head-to-tail polymerization of αβ-tubulin dimers, are involved in maintaining the cell shape and serve as tracks for axonal transport. Detached from microtubules by phosphorylation, tau may aggregate to form paired helical filaments (PHFs) and neurofibrillary tangles (NTFs) which are pathological lesions of the Alzheimer’s disease brain [4,5,6]. Tau proteins constitute a family of six isoforms which range from 352–441 amino acids. The variants differ by the presence of either three or four repeat-regions in the carboxy-terminal (*i.e.*, *C*-terminal) part of the molecule and the absence or presence of either one or two inserts in the amino-terminal part. The *C*-terminal part, known to be the microtubule binding domain, regulates the rate of microtubules polymerization [6]. Samsonov *et al.* illustrated the interaction of tau with microtubules in live neurons [7]. *In vitro*, many studies were performed to investigate the binding of tau to taxol stabilized microtubules [8,9,10]. Gustke *et al.* synthesized a variety of tau protein varying in their composition of repeat-domains and demonstrated the influence of the composition on the association parameters [9].

In a previous paper as co-authors, our laboratory has already studied the interaction of the largest tau isoform (hTau40) with taxol stabilized microtubules using cosedimentation, resonance magnetic nuclear (RMN) and fluorescence techniques [11]. By RMN spectroscopy, a molecular picture of the precise interaction zones of tau implicated in its binding to the taxol stabilized microtubule wall was described. By cosedimentation and fluorescence resonance energy transfer (FRET), binding parameters were derived. To make the FRET experiment, tau labeled with acrylodan on its two cysteines (Cys291, Cys322) has been used as acceptor of the energy of tryptophan and tyrosine residues of tubulin, as described in Makrides *et al.* [12]. FRET is an elegant tool to quantify the tau–microtubule interaction and was used by Han *et al.* to study the distances between the paclitaxel, colchicine and exchangeable GTP binding sites on tubulin using fluorescent ligands [13].

FRET, established by Förster in the 1950s [14], is the transfer between two fluorophores of the excited state energy from the initially excited donor to an acceptor. Energy transfer occurs without the emission of a photon. The transfer of energy depends on the orientation of the donor and acceptor transition dipoles, the distance between the two fluorophores, which must be within the Förster distance of 20–90 Å for biological macromolecules [15], and the spectral overlap between the donor fluorescence and the acceptor absorption. FRET, typically, leads to a reduction in the donor’s fluorescence intensity and excited state lifetime and to an increase in acceptor’s emission intensity. This technique is a useful tool to estimate distance between donor and acceptor fluorophores [16] and conformational changes in biomolecules [17]. Since FRET occurs over a distance similar to the size of proteins, it is widely used to detect protein–protein interactions in cells [18,19,20].

Herein, to investigate the binding of tau protein to the microtubules, the energy transfer between microtubules stabilized with Flutax-2 and hTau40 protein labeled with carboxy tetramethylrhodamine succinimidyl ester was measured. Flutax-2 [21] is a taxoid labeled at position 7 of the taxane ring with difluorofluorescein and is an inducer of microtubule assembly as taxol. With Flutax-2 acting as the donor and the amine reactive dye, rhodamine, as the acceptor, the distance separating derivatized tau from the fluorescent microtubules upon binding was deduced. Binding parameters were calculated from titration curves by holding the microtubule concentration constant and by varying the tau concentration.

Tau has a mostly basic character, except for *N*-terminal which is mainly acidic. Its repeat-domains and the two regions flanking the repeats are both implicated in a tight binding to microtubules [22], particularly to the *C*-terminal domain of α- and β- tubulin comprising the helices H11, H12 and the disordered acidic *C*-terminal tail [23,24]. To go deeper in our investigation, the role of the hypervariable and highly acidic *C*-terminal tails of α- and β-tubulin in the interaction with tau protein was determined. Once experimental conditions to produce a homogeneous solution of cleaved tubulin were set up, binding parameters were measured and compared to those obtained with undigested tubulin.

## 2. Results and Discussion

### Fluorescence Resonance Energy Transfer (FRET)

**Figure 1:**
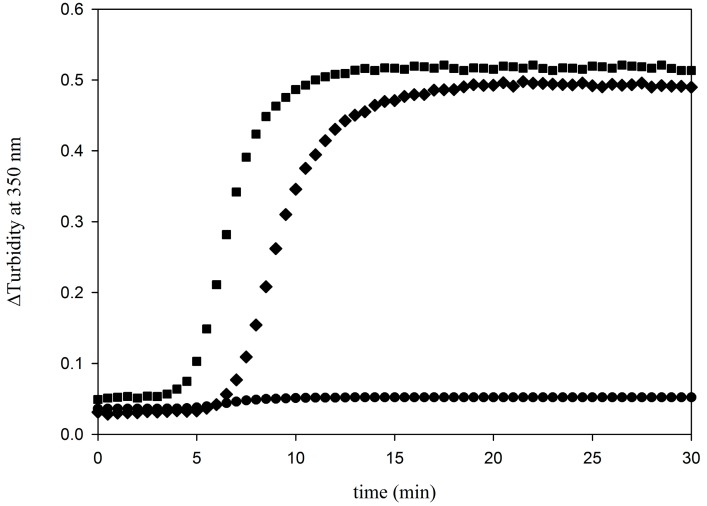
Turbidimetric assay illustrating the kinetic behavior of tubulin (10 μM) assembly at 37 °C induced by un-labeled tau (5 μM) (square symbols) and labeled with rhodamine (diamond symbols) (5 μM) in PG buffer (*i.e.*, 20 mM NaPi, 1 mM tris(2-carboxyethyl)phosphine (TCEP), 0.1 mM guanosine 5'-triphosphate (GTP), pH 6.5). Circles represent a solution of 10 μM tubulin without tau.

Labeled protein tau (*i.e.*, tau-rhodamine) was used as the acceptor in the FRET studies. Labeling was achieved in buffer with near neutral pH to specifically label the amine terminal (*i.e.*, *N*-terminal) of tau. Indeed, the p*K*a of the *N*-terminal of a peptide is lower than the p*K*a of the lysine present in the lateral chain. Before looking at the energy transfer between this species and the microtubules stabilized with Flutax-2 (*i.e.*, fluorescent microtubules), which act as the donor, the functionality of tau-rhodamine to induce microtubules formation was studied. It is well known that tau stimulates the assembly of tubulin into microtubules at tubulin concentrations below the critical concentration for tubulin self-assembly [1]. Figure 1 illustrates the kinetic analysis of a solution of 10 µM tubulin in the presence of 5 µM tau-rhodamine at 37 °C. The turbidity at 350 nm attains a steady state value after 20 min. In comparison with the time course of 10 µM tubulin alone, it is shown that tau-rhodamine, as unlabeled tau, induces microtubules formation. This is in good agreement with the works of Lu *et al.* [25] who demonstrated, by sedimentation experiment, that tau-rhodamine bound efficiently to the assembled microtubules. Identically, the turbidimetric analysis of fluorescent microtubules assembly showed an absorbance plateau after 20 min at 37 °C (data not shown). The assembly of tubulin in presence of Flutax-2 was demonstrated to be the same as the assembly with the other fluorescent taxoid, Flutax-1 [26] except for the critical tubulin concentration which is similar to taxol (*i.e.*, 1.5 µM) [21].

**Figure 2:**
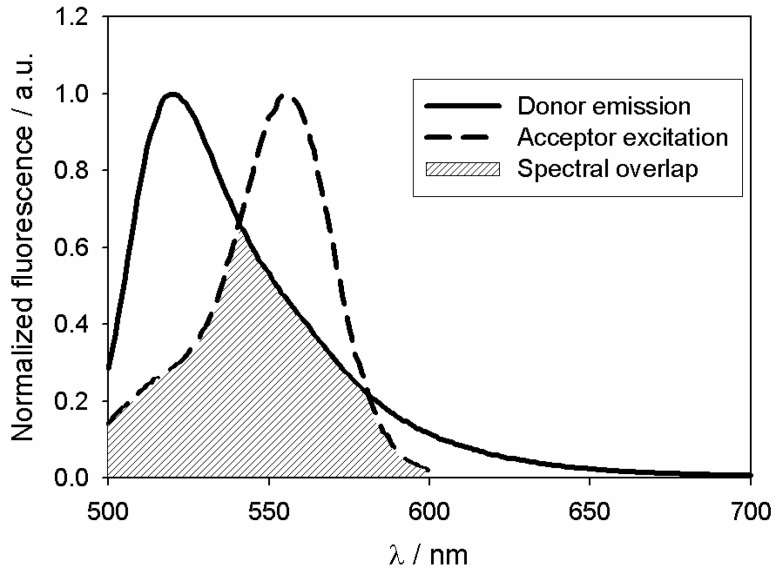
Normalized emission spectrum of Flutax-2 bound to microtubules (λ_exc_ = 494 nm, λ_em_ = 520 nm) and excitation spectrum of tau labeled with rhodamine (λ_exc_ = 556 nm, λ_em_ = 580 nm). Shaded area determines the spectral overlap between the donor emission and the acceptor absorption, the molar extinction coefficient of tau-rhodamine is 41,955 M^−1^·cm^−1^ at 556 nm.

As viewed in Figure 2, fluorescent microtubules exhibit a fluorescence emission band at 520 nm (λ_exc_ = 494 nm). The excitation wavelength of tau-rhodamine being 556 nm, considerable spectral overlap between the two fluorophores can be seen. Indeed, in FRET, the donor molecules typically emit at shorter wavelength and its spectrum overlaps with the absorption spectrum of the acceptor. The integral overlap, *J*(λ) from Equation (2), is 1.6 × 10^−13^ M^−1^·cm^3^. The fluorescence quantum yield of free Flutax-2 in absence of the acceptor was measured as 0.90 ± 0.02 in NaCl-PG buffer (λ_exc_ = 494 nm). According to Lillo *et al.* [27], the bound Flutax-2 exhibits a quantum yield identical to free Flutax-2. Indeed, at pH 7, the intensity in fluorescence of free Flutax-2 is due to its dianionic form (*pK*a = 4.83 [28]) which is the only emitting species and presents little change upon binding [21]. A value of 0.90 was therefore taken for ΦD. Assuming *η* equal to 1.4 as for biomolecules in aqueous solution and *κ*2 to 2/3, which is appropriate for donors and acceptors rotating randomly prior to energy transfer [13,27], the Förster distance, *R*_0_, is equal to 52.9 ± 0.1 Å from Equation (1). As *R*_0_ satisfies the criterion of distance between the donor and the acceptor (*i.e.*, 20–90 Å), energy transfer can take place. Energy transfer is the result of dipole–dipole interaction between the two species and can be determined by steady-state measurements of the extent of donor quenching due to the acceptor.

**Figure 3:**
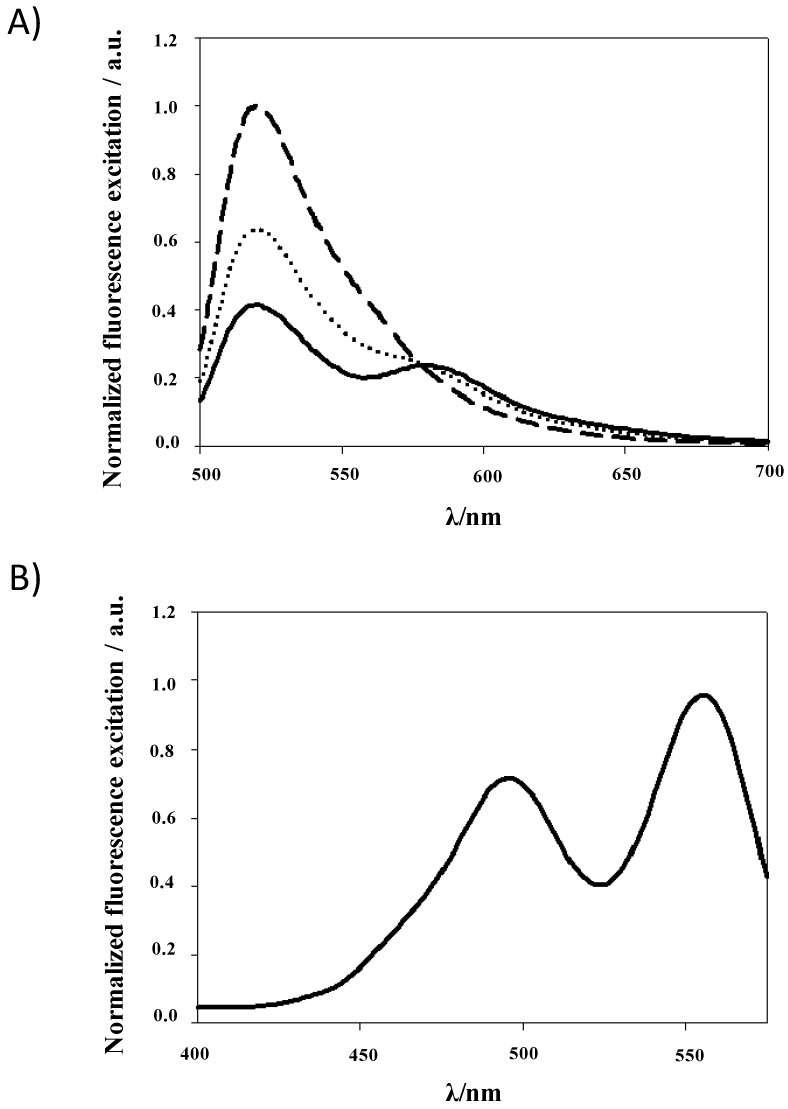
(**A**) Normalized emission spectrum of fluorescent microtubules in NaCl-PG buffer (*i.e.*, 25 mM NaPi, 25 mM NaCl, 1 mM TCEP, 0.1 mM GTP, pH 7) in absence (– –) or in presence (··· and ―) of tau-rhodamine (*i.e.*, 5 and 18 µM, respectively) recorded with an excitation wavelength of 494 nm. The tubulin concentration was 5 µM; (**B**) Normalized excitation spectrum of the complex composed of fluorescent microtubules and tau-rhodamine demonstrating energy transfer between the two fluorophores. The emission wavelength used was 580 nm.

Figure 3A illustrates the emission spectrum of fluorescent microtubules which is quenched considerably upon addition of tau-rhodamine. The peak at 580 nm in the emission spectrum of the complex reflects rhodamine fluorescence. To highlight the energy transfer, the fluorescence excitation of the complex at λ_em_ of 580 nm was recorded (Figure 3B). As said in Albani *et al.* [29], in absence of donor-to-acceptor transfer, at the maximum emission of rhodamine (*i.e.*, λ_em_ = 580 nm), the excitation spectrum should be identical to the absorption spectrum of tau-rhodamine. On the contrary, a band at 494 nm is seen corresponding to the donor excitation spectrum.

**Figure 4:**
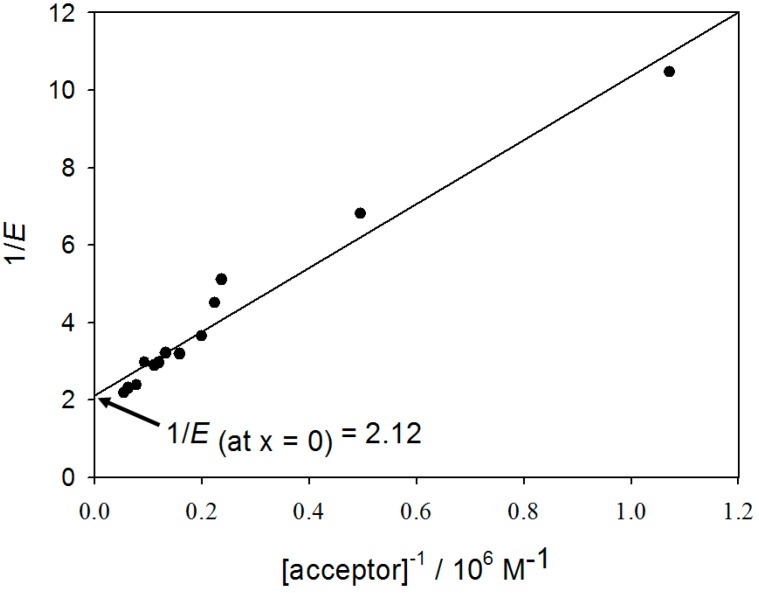
Determination of the efficiency of energy transfer, *E*, between the fluorescent microtubules and tau-rhodamine. The plot gives a straight line where the intercept with the *y* axis is 1/*E* (=2.12) at infinite concentration of acceptor.

Energy transfer efficiency, *E* from Equation (3), was determined by linear extrapolation to acceptor concentration zero using the fluorescence intensity values of the donor at 520 nm corrected for the inner filter effect (Equation (5)).The molar extinction coefficient used are 8624 M^−1^·cm^−1^ at 494 nm (λ_exc_) and 20,983 M^−1^·cm^−1^ at 520 nm (λ_em_). A plot of 1/*E* *versus* 1/[acceptor] gives a FRET efficiency of 47% ± 3% (Figure 4). The distance, *r*, between the donor and the acceptor was then calculated to be 54 ± 1 Å. Data are summarized in Table 1 (using MT-(Flutax-2) as an abbreviation for Flutax-2 bound to microtubules).

**Table 1:** Calculated parameters obtained from the spectral analysis of the protein mixture.

Donor	Acceptor	*E* [%]	*R*_0_ [Å]	*r* [Å]
MT-(Flutax-2) ^a^	Tau-rhodamine ^b^	47 ± 3	52.9 ± 0.1	54 ± 1

^a^ λ_exc_ = 494 nm, λ_em_ = 520 nm; ^b^ λ_exc_ = 556 nm, λ_em_ = 580 nm.

**Figure 5:**
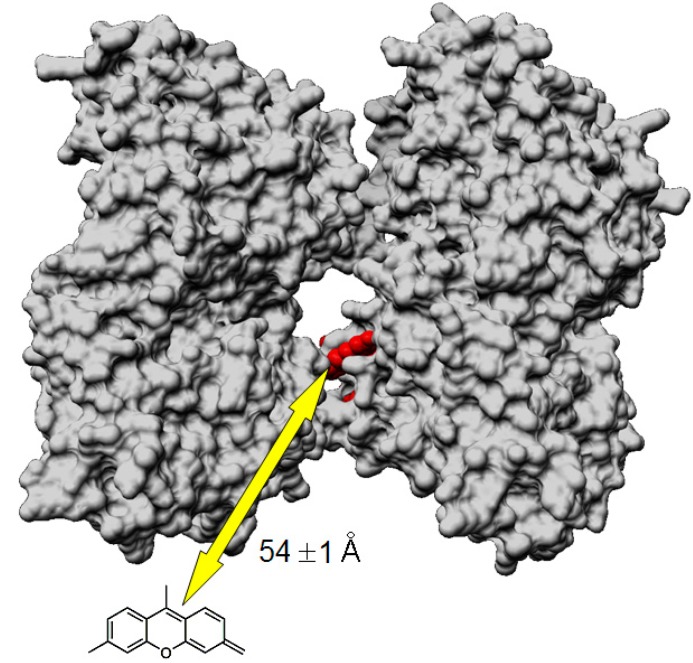
Van der Waals representation of the outer surface of a fragment (two tubulin dimers) of a high resolution microtubule model. Flutax-2 is shown in red between the two tubulin subunits. Tau is illustrated by its fluorescence moiety, rhodamine. The arrow is a radius of a sphere centred on the donor (*i.e.*, bound Flutax-2) on which the acceptor (*i.e.*, tau-rhodamine) has been found. This radius is equal to 54 ± 1 Å.

Figure 5 portrayed a model microtubule wall where Flutax-2 is shown in red inside the pore between two tubulin dimers [30]. As the molecular 3D structure of tau protein remains unknown [31], tau is represented by its fluorescent moiety (*i.e.*, 5,6-TAMRA) corresponding to its *N*-terminal domain. The arrow illustrates the radius (*i.e.*, 54 ± 1 Å) of a sphere in which the centre is the Flutax-2 molecule. The position of Flutax-2 allows direct accessibility for energy transfer to the bulk of the solution and in particular to tau-rhodamine molecules. We studied the binding of derivatized tau on preformed microtubules stabilized by Flutax-2. In these conditions, it was admitted that tau binds to the outer surface of the microtubule wall [8,23,24,32]. As a consequence, the inner microtubule surface of the sphere could be omitted. NMR study [11] showed that the four microtubules binding domain become immobilized on the microtubule surface and that the *N*-terminal domain of tau projected from it. More recently, Magnani *et al.* [33] demonstrated that *N*-terminal part of tau was implicated in the binding of the *C*-terminal domain of P150 subunit of dynactin complex. Using electron microscopy and immunoelectron microscopy, they proposed a model where the tau projection domain was at approximately 40 Å from the microtubule wall. Their finding argues for the distance found by FRET in this study.

## 3. Determination of the Binding Parameters

**Figure 6:**
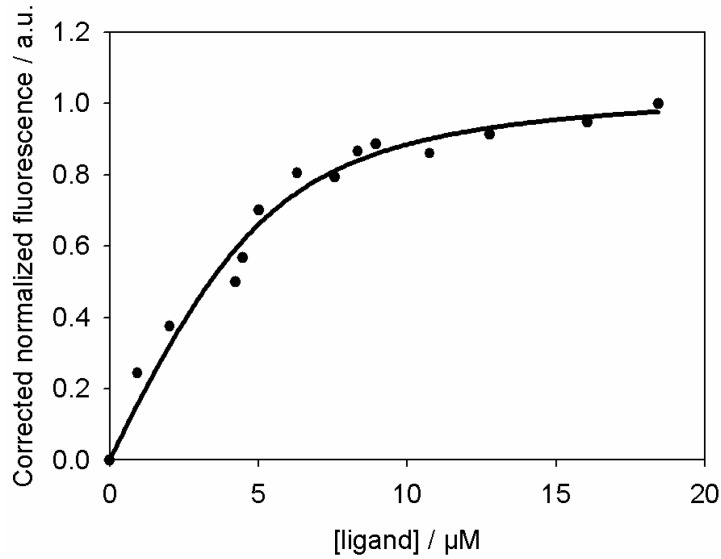
Fluorescence quenching titration produced by the binding of tau to the microtubules at 520 nm. Fluorescence values were corrected for the inner filter effect (Equation (5)) and the titration curve inverted. The titration was done against a fixed concentration (5 µM) of microtubules. The apparent dissociation constant, calculated from Equation (6), is 1.0 ± 0.5 µM, setting the stoichiometry, *n*, to 1.

First, we examined tau–microtubule interaction, from the quenching of fluorescence observed at 520 nm during the FRET experiment (Figure 3A), the binding parameters of the tau–microtubule interaction were derived. Figure 6 shows the titration curve for the association of tau-rhodamine to fluorescent microtubules. The apparent stoichiometric dissociation constant (*K_d_*) was fitted assuming a single class of sites and was found to be equal to 1.0 ± 0.5 µM. Similar values were found by titrating the increase in differential fluorescence at 556 nm (data not shown). The way of calculating differential fluorescence is explained in a previous paper [34]. The calculated apparent dissociation constant is consistent with published values [9,35] and with the cosedimentation experiment carried out in our laboratory [11]. However, affinity constants, reported in the literature, varied from 0.1 µM to above 1 µM [8,10]. This variation depends strongly on experimental procedures. For example, Butner *et al.* titrated fixed tau against an excess of microtubules and detected bound tau by radioactivity of incorporated 35S [35]. Gustke *et al.* used fixed microtubule concentration, varied tau in the µM range, and analyzed the fractions by quantifying SDS gels [9]. Ackmann *et al.* titrated tau against different fixed microtubule concentrations and developed an ELISA assay to detect tau [8]. The apparent dissociation constant, obtained by FRET with the fluorophore acrylodan, was found to be in the nanomolar range with a stoichiometry close to 0.40 [11,12]. The fast dissociation of tau from taxol-stabilized microtubules is inconsistent with a tight binding of tau to microtubules. This could correspond to a population which presents a reversible binding of tau to the microtubule surface during the first phase of interaction, the second phase being the accumulation of tau onto the microtubule surface. All these discrepancies between the reported values suggest that the tau–microtubule interaction is complex because of the natively unfolded structure of tau allowing it to adopt different conformation upon binding. The influence of the modification of tau due to its phosphorylation or oxidation increases the level of difficulty in understanding the molecular mechanism of tau-microtubule binding.

Our laboratory has previously demonstrated that the tau phosphorylation of the serine 214 by the PKA kinase decreases the strength of tau-microtubule interaction by two orders of magnitude and the presence of an intramolecular disulfide bridge, on the contrary, led to a partial detachment of the *C*-terminal of tau, and decreased significantly the overloading of tau on the microtubule surface [11]. Another candidate which could have a major influence on the tau-microtubule interaction is the *C*-termini of α- and β- tubulin which is exposed to the outer surface of microtubules and therefore to tau protein. In the literature, some authors affirm that the absence of the *C*-terminal of αβ-tubulin inhibits the binding of tau to the microtubules [36] or that the binding parameters are reduced by 15% (cosedimentation experiment) [32]. Santarella *et al.* [37] showed that the absence of the *C*-terminal of β-tubulin does not change the tau binding. On the other hand, when both α- and β-tubulin are cleaved, tau does not bind anymore.

**Figure 7:**
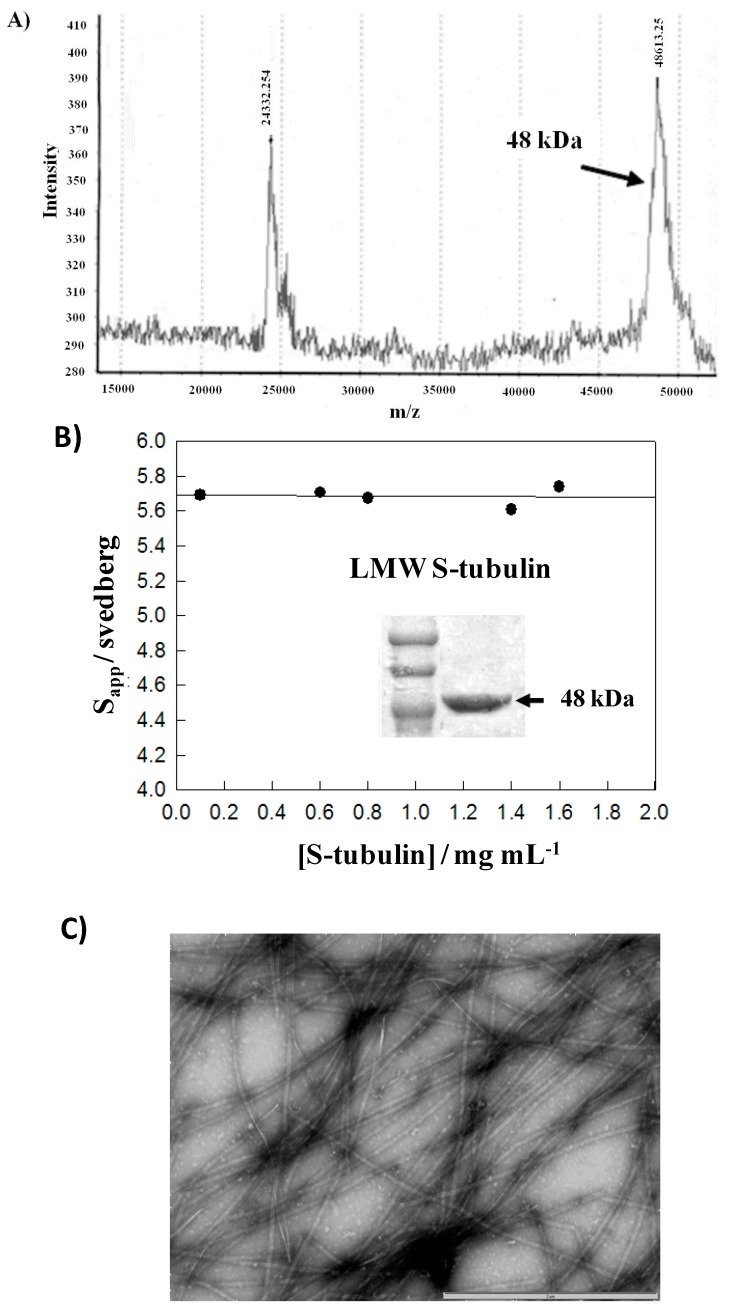
Subtilisin cleaved tubulin characterization: (**A**) Mass spectrum of S-tubulin showing one species of 48 kDa identified after MALDI-TOF/MS analysis. Monomer of 24 kDa is given; (**B**) Determination of the S-tubulin sedimentation coefficient by analytical ultracentrifugation. After extrapolation to protein zero concentration, S°_20,w_ equal to 5.69 ± 0.05 S. In inset, the SDS-PAGE analysis of S-tubulin gives one major band at 48 kDa. Low molecular weight markers (LMW) are from top to bottom 97, 66 and 45 kDa; (**C**) Electron microscopy of microtubules after assembly of S-tubulin induced by taxol with a molar ratio of 1:1 in NaCl-PG buffer (*i.e.*, 25 mM NaPi, 25 mM NaCl, 1 mM TCEP, 0.1 mM GTP, pH 7). The bar represents 2 µm.

Second, this issue leads to the technical developments of homogenous samples of cleaved tubulin. *C*-terminal cleavage of tubulin by subtilisin treatment was obtained by the procedure of Kanazawa and Timasheff [38] modified as described in the Experimental Section. Subtilisin cleaves α and β monomers of tubulin at their *C*-termini as they present a high charge density. The cleaved protein is referred to as S-tubulin or αS βS and was characterized by MALDI―TOF mass spectrometry (Figure 7A). The mass spectrum shows only two peaks, at 48,613 and 24,332 Da. The latter corresponds to the 2+ charge state of S-tubulin. No trace of 35,000 and 21,000 Da peptides described as proteolysis degradation products [39] was observed indicating a pure sample of S-tubulin. Therefore, both α- and β-tubulin subunits were cleaved. Subtilisin and *C*-terminal tubulin fragments were totally discarded with this method. It is of great importance to remove the negatively charged amino acids from the tubulin subunits as they decrease their capacity for self-assembly in terms of charge repulsion [40,41,42]. Moreover, analytical centrifugation velocity experiments at various S-tubulin concentrations were carried out to determine the standard sedimentation coefficient (S°_20,__w_) of S-tubulin. After determination and correction to standard conditions (20 °C in water), the apparent coefficients were plotted *versus* protein concentrations (Figure 7B). The linear extrapolation to protein concentration zero gives a value of 5.69 ± 0.05 S corresponding to the S°_20,w_. For tubulin, S°_20,w_ is 5.8 S, the 0.1 S loss was in agreement with a loss of molecular mass. SDS-PAGE analysis of S-tubulin followed by Coomassie Brilliant Blue staining revealed a band at 48 kDa as observed in the mass spectrometry analysis part (Figure 7A). It was previously shown that the assembly product from S-tubulin consisted mainly of protofilament bundles, hooked polymer, and open tubules [40] or rings [43] as a function of experimental conditions. After such observations, electron microscopy analysis of the assembled microtubules from S-tubulin stabilized with taxol at a mixing molar ratio of 1:1 in NaCl-PG buffer was made and shows a regular assembly of long microtubules (Figure 7C).

Third, the interaction between tau-rhodamine and S-tubulin-microtubule was studied by FRET experiment titration (data not shown), a *K_d_* value of 3.1 ± 1.5 µM was found. The removal of the *C*-terminal region revealed a three-fold decrease in the apparent binding constant previously found for undigested tubulin (*i.e.*, 1.0 ± 0.5 µM, see above). Even if the energy transfer Förster and donor-to-acceptor distances gave similar results than those presented in Table 1 for undigested tubulin, we cannot assert that the *C*-termini of αβ-tubulin chains are not involved in tau binding onto stabilized microtubules, as suggested by Saoudite *et al.* [44]. Our binding results indicate that the decrease in negative charges caused by the removal of portions of the acidic *C*-termini of α- and β-tubulin chains reduces slightly the interaction of a positively charged macromolecule as tau. The role of the carboxy-terminal regions is more complex than the classically electrostatic repulsive or attractive effect reported. Indeed, in the 3D structure determination by electronic or X-rays diffraction, the *C*-termini of tubulin could not be resolved because it is highly flexible and lies outside the globular domain [45,46]. In 2005, Priel *et al.* modeled the dynamical states of the *C*-termini [47]. In this model, each *C*-termini can either bind to the tubulin surface via one of the several positively charged regions or more probably can explore the space available in the solution. This space exploration function for the *C*-termini tails of tubulin would permit or facilitate the recruitment of protein interacting with microtubules. This also explains the increase of the affinity with undigested tubulin. However, the decrease observed for *K_d_* is not drastic, hence tau interacts largely with unknown regions of tubulin.

## 4. Experimental Section

### 4.1. Materials

7-*O*-[*N*-(2,7-difluoro-4'-fluoresceincarbonyl)-l-alanyl]Taxol (Flutax-2) [21], was a gift of José. M. Andreu, Centro de Investigaciones Biológicas, CSIC, Madrid, Spain. 5-(and-6) carboxy tetramethylrhodamine succinimidyl ester (5(6)-TAMRA) was supplied from Molecular Probes (Eugene, OR, USA). Vivaspin 500 µL centrifugal filters were from Sartorius Stedim Biotech SA (Aubagne, France). Sephadex G-25 and G-50 media were purchased from GE Healthcare (Uppsala, Sweden), preswollen microgranular diethylaminoethyl Cellulose (DE-52) from Whatman plc (Kent, UK). All other chemicals were from Sigma Chemicals Co. (St Louis, MO, USA).

Tau protein was purified on an ÄKTATM purifier from GE Healthcare. Absorbance spectra were recorded on a Perkin-Elmer (Waltham, MA, USA) LambdaTM 800 UV/Vis spectrophotometer, fluorescence excitation and emission spectra on a FluoroMax^®^-3 from Horiba Jovin Yvon (Edison, NJ, USA) with slit widths of 1/1 nm, using 1 cm (excitation direction) × 0.2 cm (emission direction) cells (Hellma) thermostated at 25 °C.

### 4.2. Tau Purification and Labeling

hTau40 was expressed from a pET vector introduced into Escherichia coli BL21(DE3) as previously described [48], and suspended in a purification buffer (*i.e.*, 50 mM 2-(*N*-Morpholino)ethanesulfonic acid (MES), 0.4 mM dl-dithiothreitol (DTT), 1 mM phenylmethylsulfonyl fluoride (PMSF), 5 mM (Ethylenedinitrilo)tetraacetic acid (EDTA), 1 protease inhibitor tablet, 1% triton^®^-X-100, pH 6.5). The lysate was then cleared by centrifugation at 12,000 rpm (Sorvall^®^-RC28S centrifuge) for 20 min at 4 °C, after sonication (15 µL, 3 × 2 min) and heating at 90 °C. The supernatant was loaded onto a 5 mL HiTrapTM SP Sepharose HP cation exchange column (GE Healthcare) firstly equilibrated with 50 mM MES (pH 6.5) and eluted with a buffer consisting of 50 mM MES and 0.5 M sodium chloride (NaCl) (pH 6.5). Further purifications were achieved by using a reverse-phase column (SourceTM 15RPC PE 7.5/150, GE Healthcare) pre-equilibrated with H2O/trifluoroacetic acid (TFA) (0.065% *v*/*v*). After elution with Acetonitrile/TFA (0.05% *v*/*v*), fractions containing tau protein were pooled and dry-lyophilized.

Before derivatization of purified tau with 5(6)-TAMRA [25], powder was resuspended in labeling buffer (*i.e.*, 20 mM sodium phosphate (NaPi), 1 mM tris(2-carboxyethyl)phosphine (TCEP), pH 6.5) and tau concentration measured at 280 nm using a molar extinction coefficient of 7700 M^−1^·cm^−1^. 5(6)-TAMRA, freshly dissolved in dimethyl sulfoxide (DMSO), was added to tau at a dye:protein molar ratio of 35:1. The mixture was immediately vortexed and left in the dark for 2 h. The free dye was removed by passing the derivatized solution through a 5 mL HiTrapTM desalting column (GE Healthcare) pre-equilibrated with the labeling buffer. The concentration of tau conjugated with 5(6)-TAMRA was determined by absorbance at 280 nm after correction of the amount of absorbance caused by the dye. The degree of labeling [49] was calculated using a molar extinction coefficient of 41,955 M^−1^·cm^−1^ at 556 nm and estimated to be close to 1 mole of dye per mole of protein. Labeled tau was further concentrated onto Vivaspin 500 µL centrifugal filters.

### 4.3. Tubulin Purification and Microtubule Assembly

Tubulin was extracted and purified from lamb brains by Weisenberg procedure consisting in ammonium sulfate fractionation and ion exchange chromatography [50,51]. Aliquots were stored in liquid nitrogen.

Before assembly, tubulin was equilibrated as described [43,52]. Briefly, protein was collected after passage through cold sephadex G-25 columns pre-equilibrated with NaCl-PG buffer (*i.e.*, 25 mM NaPi, 25 mM NaCl, 1 mM TCEP, 0.1 mM guanosine 5'-triphosphate (GTP), pH 7) and the tubulin concentration determined spectrophotometrically at 275 nm using a molar extinction coefficient equal to 109,000 M^−1^·cm^−1^ in 6 M guanidine hydrochloride or 107,000 M^−1^·cm^−1^ in 0.5% sodium dodecyl sulphate (SDS).

Fluorescent microtubules were assembled by adding 7 µM Flutax-2 and 7 mM magnesium chloride (MgCl2) to a 5 µM tubulin solution and by raising the temperature to 37 °C for 25 min. The reaction was followed by plotting the turbidity at 350 nm, which is proportional to the weight concentration of long microtubules [53], as a function of time, and terminated when the absorption values reached the microtubule assembly plateau.

### 4.4. C-Terminal Cleavage of Tubulin

Limited proteolysis with subtilisin from bacterial type XXIV (0.8% *w*/*w*) was carried out in PMG buffer (*i.e.*, 20 mM NaPi, 1 mM MgCl2, 0.1 mM GTP, pH 7) for 15 min at 25 °C. Digestion was stopped with the addition of 1 mM PMSF and the solution incubated in ice for 10 min. Sample was mixed with 2 mL of DE-52 anion exchange resin in a centrifuged column firstly equilibrated with PMG buffer and left 10 min in ice. The column was then washed with 5 mL of PMG buffer and centrifuged at 3000 rpm (GR4.11 Jouan centrifuge) for 7 min to eliminate the buffer. To elute cleaved tubulin, the resin was resuspended in 0.38 M NaCl (final concentration), incubated 5 min at 4 °C and centrifuged at 3000 rpm during 7 min. Filtrate was passed sequentially through a G-25 (0.9 × 13 cm) and a G-50 (0.9 × 20 cm) gravity columns, pre-equilibrated with PMG and NaCl-PG buffer, respectively. Protein concentration determination and microtubule assembly with Flutax-2 were carried out as for the tubulin which was not cleaved with subtilisin.

### 4.5. Mass Spectrometry Experiments

Positive ion mass spectral analysis of cleaved tubulin was carried out by matrix laser assisted desorption ionization-time-of-flight (MALDI-TOF) in linear mode using a MALDI-TOF Ettan-Pro (GE Healthcare, Amersham, England, UK) mass spectrometer equipped with delayed extraction. Prior analysis, proteins (1 picomole·µL^−1^) were mixed in a 1:1 (*v*/*v*) ratio with a saturated solution of sinapinic acid (LaserBio labs, Sophia-Antipolis, France) in 0.1% aqueous TFA. External mass calibration was performed using horse heart myoglobin and bovine serum albumin.

### 4.6. Sedimentation Velocity Experiments

The experiments were carried out at 40,000 rpm and 20 °C on a Beckman (Fullerton, CA, USA) Optima XL-A analytical ultracentrifuge equipped with absorbance optics, using a four An55Ti and a eight holes An50Ti rotors and 1.2 cm Epon double-sector centerpieces. Samples of cleaved tubulin were prepared in PG buffer (*i.e.*, 20 mM NaPi, 0.1 mM GTP, pH 6.5). Apparent sedimentation coefficients, *S*app, were determined by the sedimentation coefficient distribution *C*(S) generated by SEDFIT program [54] and corrected to standard conditions with SEDNTERP program.

### 4.7. Cosedimentation Assay and Polyacrylamide Gel Electrophoresis

The assay was performed on cleaved tubulin as described in Sillen *et al.* [11] Polyacrylamide gel electrophoresis in denaturing conditions (SDS-PAGE) was performed using 12% acrylamide in the separating gel and GE Healthcare low-weight calibration kit (97, 66, 45, 30, 20.1, and 14.4 kDa) for standards. Gels were stained with Coomassie brilliant Blue.

### 4.8. Electron Microscopy

Before analysis, microtubules, obtained from cleaved tubulin and induced by taxol in a ratio 1:1 in NaCl-PG buffer, were absorbed onto 200 mesh Formvar carbon-coated copper grids (Canemco Inc. & Marivac Inc., Lakefield, QC, Canada) stained with 1.5% (*w*/*v*) uranyl acetate, and blotted to dryness. Grids were observed onto a JEOL (Tokyo, Japan) JEM-1220 transmission electron microscope operated at 80 kV.

### 4.9. Fluorescence Resonance Energy Transfer (FRET)

The Förster distance between the donor and acceptor sites (*R*_0_), which is the distance at which the energy transfer efficiency of FRET is 50%, in angstroms (Å), is given by [15]:

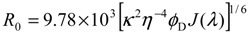
(1)
where *κ*^2^ is the factor describing the relative orientation between the transition dipole of donor and that of the acceptor, *η*, the refractive index of the medium, *Φ*_D_, the quantum yield of the donor in the absence of the acceptor. The integral, *J*(*λ*), expresses the degree of spectral overlap between the donor fluorescence emission and the acceptor absorption as described below:

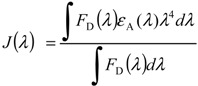
(2)
where *F*_D_ is the donor fluorescence intensity, *ε_A_*, the acceptor extinction coefficient and *λ*, the wavelength.

According to Förster, the efficiency (*E*) of FRET process depends on the inverse sixth-distance between donor and acceptor (*r*) as well as on the critical energy transfer distance or Förster radius (*R*_0_) under the condition of 1:1 situation of donor: acceptor concentrations. E represents the fraction of photons absorbed by the donor that are transferred to the acceptor and is typically measured using the relative fluorescence intensity of the donor in absence (*F*_D_) and presence (*F*_AD_) of the acceptor:

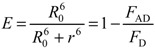
(3)

Quantum yield of the donor in the absence of the acceptor was determined experimentally. Fluorescein disodium was used as standard with a quantum yield of 0.92 ± 0.02 in 0.1 N NaOH [55,56]. The general equation for the determination of relative quantum yield, *Φ*, is expressed as:

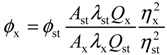
(4)
where subscripts x and st refer to the unknown and the standard, respectively. The parameter *A* is the absorbance at the excitation wavelength, *λ* and *Q* is the integrated area under the fluorescence emission spectrum. The square of the refractive index, *η*, is employed to make the correction due to difference in media of the unknown and the standard.

### 4.10. Determination of the Binding Parameters

Energy transfer between fluorescent microtubules and tau derivatized with 5(6)-TAMRA was followed by monitoring the decrease in fluorescence emission at 520 nm upon addition of labeled tau. After correction for the inner filter effect (Equation (5)) [15], the normalized fluorescence intensity values at 520 nm were plotted *versus* ligand concentrations.

*F*_corr_ = *F*_obs_·10^(*A*_exc_ + *A*_em_)/2^ (5)

where *F*_corr_ is the fluorescence intensity after correction, *F*_obs_, the experimental fluorescence intensity, *A*_exc_ and *A*_em_ refer to the absorption of the ligand at the excitation and the emission wavelengths, respectively (the *A*_em_ was corrected to account for the 0.2 cm pathlength).

The binding parameters of Equation (6) were fitted as described [34], using nonlinear least-square curve fitting program Sigmaplot^®^.


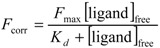
(6)
where *F*_max_ is the plateau fluorescence value, *K_d_* is the apparent stoichiometric dissociation constant of the interaction between the microtubules and the ligand (*i.e.*, labeled tau protein). All experiences are done in triplicate, permitting calculation of a mean value and the standard deviation.

## 5. Conclusions

In the present work, the quantification of the interaction of tau to microtubules using FRET is presented. The distance separating the microtubules stabilized with Flutax-2 and tau derivatized with 5,6-TAMRA (*i.e.*, tau-rhodamine) was found to be 54 ± 1 Å at 47% efficiency. This seems to indicate that tau binds mainly to the external part of the microtubule wall and near the pore regions. The binding parameters, calculated after titration of fixed microtubule concentration with an excess of tau, gave a *K_d_* value of 1.0 ± 0.5 µM for the apparent stoichiometric dissociation constant. The *C*-terminal tails of αβ-tubulin, which contains the last amino acids of the α- and β-tubulin chains after the helices H11 and H12, has shown no change in the calculated donor-to acceptor distance. However, the decreased affinity parameter suggests that the *C*-terminal regions of αβ-tubulin could be slightly involved in tau binding by electrostatic and recruitment effects.
